# Advancing the science of distress screening and management in cancer care

**DOI:** 10.1017/S2045796019000799

**Published:** 2020-01-09

**Authors:** Kristine A. Donovan, Luigi Grassi, Teresa L. Deshields, Cheyenne Corbett, Michelle B. Riba

**Affiliations:** 1 Moffitt Cancer Center, Tampa, FL, USA; 2 Institute of Psychiatry, University of Ferrara, Ferrara, Italy; 3 Rush University Cancer Center, Chicago, IL, USA; 4 Duke Cancer Institute, Durham, NC, USA; 5 University of Michigan Rogel Cancer Center, Ann Arbor, MI, USA

**Keywords:** Mental health, oncology, psychiatric services, psychological assessment

## Abstract

Given the high prevalence (30–35%) of psychosocial and psychiatric morbidity amongst cancer patients in any phase of the disease trajectory, screening for emotional problems and disorders has become mandatory in oncology. As a process, screening begins at the entry to the cancer care system and continues at clinically meaningful times, periodically during active cancer care, or when clinically indicated. The goal is to facilitate proper referral to psychosocial oncology specialists for more specific assessment and care, as well as treatment and evaluation of the response, according to the implementation of distress management guidelines. In this editorial, we will provide a non-exhaustive overview of relevant protocols, with particular reference to the National Comprehensive Cancer Network (NCCN) Distress Management in Oncology Guidelines, and review the challenges and the problems in implementing screening, and the assessment and management of psychosocial and psychiatric problems in cancer centres and community care.

## Introduction

A number of studies have shown that people with cancer experience a vast array of needs, not only in the physical, informational, practical and social domains, but also in the emotional and psychological domains. Indeed, all phases of the cancer trajectory, from diagnosis to long-term survival, from recurrence to advanced disease and end of life, are *foci* for the development of psychological problems or, in 30–35% of cancer patients, more specific psychiatric disorders (Mitchell *et al*., 2011). As indicated in the Institute of Medicine report (IOM, 2008), a comprehensive understanding of patient-specific psychosocial health care needs is an essential precursor to appropriate interventions and for facilitating access to relevant psychosocial and supportive care services. Screening for emotional well-being has thus become standard of care in oncology and involves (1) screening at system entry, clinically meaningful events, periodically during active cancer care or other stressful times, (2) referral to appropriate psychosocial oncology specialists for assessment and care, and (3) treatment and evaluation of the treatment response according to the implementation of distress management protocols (Smith *et al*., 2018). Today, comprehensive clinical guidelines for screening and management of distress and psychosocial needs are available in some countries, such as Australia. These documents have been published with the aim of helping clinicians address the psychosocial needs of their patients by using evidence- and consensus-based practice guidelines (Turner, 2015; Butow *et al*., 2015). Likewise in Canada, standards of care, organisational standards, educational standards and clinical guidelines for the screening, assessment and care of psychosocial distress in all phases of the cancer trajectory have been published by the Canadian Partnership Against Cancer and the Canadian Association of Psychosocial Oncology (Howell *et al*., 2015).

## National Comprehensive Cancer Network (NCCN) distress management in oncology

In the USA, the NCCN Distress Management Panel, consisting of multidisciplinary health care professionals, has also developed guidelines on distress screening and management. The first edition of the guidelines was published in 1997 and the most recent version in the fall of 2019 (Riba *et al*., 2019).

The word ‘distress’ was chosen by the panel to avoid stigmatisation and to facilitate measurement by self-report. The guideline defines distress as

‘a multifactorial unpleasant emotional experience of a psychological (cognitive, behavioral, emotional), social, spiritual, and/or physical nature that may interfere with the ability to cope effectively with cancer, its physical symptoms and its treatment. Distress extends along a continuum, ranging from common normal feelings of vulnerability, sadness and fears to problems that can become disabling, such as depression, anxiety, panic, social isolation and existential and spiritual crisis.’

The Distress Thermometer (DT), a brief 0–10 visual analogue scale was developed for the purpose of routinely assessing distress in cancer patients. Possible problems (Problem List, PL) in multiple domains – including physical, emotional, spiritual, family and practical – are also assessed. The DT/PL has rapidly become one of the most widely used and validated instruments in oncology. In Canada, distress is identified as the ‘sixth vital sign’, with the same importance as blood pressure, temperature, heart frequency, breath and pain (Bultz and Carlson, 2005). Data have shown that although cut-off scores for caseness on the DT vary by language, country, clinical setting and sample characteristics, in the majority of studies, a score of ⩾4 maximises sensitivity and specificity relative to an established criterion (e.g. a psychiatric interview or other self-report measures) for psychosocial morbidity (Donovan *et al*., 2014). Policies about the routine use of the DT have been implemented in several countries as a way to identify patients reporting psychosocial morbidity and to facilitate their proper referral to psychosocial oncology services (Holland, 2013).

Since 1999, the NCCN Distress Management Panel has published the standards, updated annually, for the psychosocial care of cancer patients, establishing a set of quality measures for screening and algorithms for managing distress and psychiatric disorders (e.g. adjustment disorders, depression, suicide and suicide risk, cognitive disorders). These Guidelines for Distress Management are intended to assist clinicians in caring for cancer patients with psychosocial distress. They provide guidance on identifying patients with moderate or severe distress who require referral to psychosocial resources and on interventions for patients with mild distress. They also provide guidance for social workers, certified chaplains and mental health professionals by describing evidence-based treatments for distress.

The aim of the guidelines is that distress should be recognised, monitored, documented and treated promptly at all stages of disease and in all settings; that screening should identify the level and nature of the distress; and that ideally, patients should be screened for distress at every medical visit as a hallmark of patient-centred care, with assessment and management of distress according to clinical practice guidelines. Patients, families and treatment teams should be informed that distress management is an integral part of total medical care and be provided with appropriate information about psychosocial services in the treatment centre and the community.

The guidelines also state that interdisciplinary institutional committees should be formed to implement standards for distress management and that educational and training programmes should be developed to ensure that health care professionals and certified chaplains have knowledge and skills in the assessment and management of distress. Finally, clinical health outcomes measurement should include the assessment of the psychosocial domain (e.g. quality of life and patient and family satisfaction) and quality of distress management programmes/services should be included in institutional continuous quality improvement projects (Riba *et al.*, 2019).

In the 20 years since the initial publication of the guidelines, the literature on the prevalence and predictors of psychosocial distress, including diagnosable psychiatric disorders, and the utility of distress screening measures has grown exponentially. However, for many years, knowledge translation of the guidelines focused predominantly on identifying and measuring distress. Surveys of NCCN institutions have documented modest progress over time in the acceptance of routine distress screening as part of oncologic care and wide variability in the who, how and when of screening (Jacobsen and Ransom, 2007; Donovan and Jacobsen, 2013; Donovan *et al*., 2019). Research has clearly established that screening for distress alone is not sufficient for effectively managing distress (Mitchell, 2013). Increasingly, knowledge translation of the guidelines focuses on implementing comprehensive screening protocols with a focus on referral and treatment (Zebrack *et al*., 2015).

## From screening to referral

Beyond the initial distress screen, it is necessary to provide further assessment and triage of a positive screen in real time, referrals to identified professionals and resources, evidence-based treatment and intervention, and follow-up screening with the distressed patient (Funk *et al*., 2016). Just as with screening, the triage system must be tailored to the specific institution, clinical setting and the needs of the patient population, and must consider available resources. To date, research has documented a wide range of adherence to comprehensive screening protocols across US cancer centres with the highest rates in community cancer programmes and the lowest in NCI-designated cancer centres (Zebrack *et al*., 2017).

In the NCCN guidelines, multiple algorithms illustrate triage systems intended to support the clinical practice of providers. These algorithms recommend that all patients undergo brief psychosocial distress screening to elucidate the nature of distress, and then appropriate treatment and intervention by qualified providers. More often than not, with support and guidance as necessary, the primary oncology team can accurately assess and address mild psychosocial distress. Diagnosable psychiatric disorders, arguably often associated with moderate-to-severe distress, require further clinically appropriate assessment. The algorithms in the guidelines focus on psychiatric disorders and address these specific sources of moderate–to-severe psychosocial distress by recommending a referral to mental health professionals for evaluation, treatment and follow-up that includes communication and collaboration with the primary oncology team. The guidelines highlight the need for treatments and interventions that are relevant to the nature of the distress identified and the potential for individualised referral processes to increase the uptake of services. Most of the treatment recommendations represent expert consensus based on lower-level evidence such as clinical experience. Higher-level evidence, such as that derived from randomised controlled intervention trials, may be from other/non-cancer patients as the evidence base for treating psychiatric disorders is more extensive in the general population.

The algorithms for depressive disorders are a useful illustration of how the guidelines are organised. Depressive disorders in cancer include cancer-related depression, major depressive disorder and persistent depressive disorder. Left unaddressed, these disorders may adversely affect quality of life, treatment adherence and survival (Smith, 2015). If a patient displays any of the signs and symptoms of depressive disorders, the initial recommendation is further evaluation, diagnostic studies and modifications of related factors, for example, concurrent medications, medical causes other than cancer and a myriad of symptoms such as pain, anorexia, sleep disruption and demoralisation. This also includes assessing patient safety, the family and home environment, and alcohol and recreational drug use. In terms of assessment measures, the Patient Health Questionnaire-9 or -2 is recommended. Based on the findings, treatment approaches hinge on whether the patient is a danger to self or others. If there is no imminent danger present, recommended treatments include psychotherapy and psychotropic medication (this is supported by high-level evidence). Providers are also recommended to consider referral to social work services or chaplaincy before follow-up and re-evaluation. Although specific forms of psychotherapy or specific psychotropic medications are not identified in the algorithms themselves, in the discussion section of the current guidelines, there is an evidence-based review of specific forms of treatment by mental health professionals, including cognitive behavioural therapy, supportive psychotherapy and family and couples therapy. Similarly, a review of treatment with antidepressant medication and/or anxiolytic medication is included. The focus of the algorithms in the guidelines is to improve the quality of care by integrating psychosocial care into routine oncologic care. To this end, algorithms are available for the neurocognitive disorders dementia and delirium, depressive disorders, bipolar and related disorders, schizophrenia spectrum and other psychiatric disorders, anxiety disorders, trauma and stress-related disorders (including adjustment disorders), obsessive–compulsive and related disorders, substance-related and addictive disorders, and personality disorders. When appropriate, an aspect of the guidelines is linked to other guidelines to harmonise and minimise duplication across the various NCCN guidelines for supportive care; as for example, when the identification of fatigue as a problem is linked to the Guidelines for Cancer-Related Fatigue or a sexual health problem is linked to the Guidelines for Survivorship.

## Usefulness of the distress management guidelines in oncology

With respect to referral processes, a recent systematic review by McCarter *et al*. (2018) illustrates the discrepancy between actual practice and guideline recommendations for referral of distressed patients for further assessment and treatment by mental health professionals. The review identified just five studies, with predominantly poor methodological quality, that examined the effectiveness of strategies to improve the implementation of distress screening and referral. In conclusion, the authors noted the paucity of evidence for strategies to improve rates of referral to psychosocial support and treatment and the need to establish a strong evidence base supporting the implementation of comprehensive distress screening protocols.

Research indicates that many patients, even when identified as distressed, tend to decline help (Clover *et al*., 2015). A study by Tondorf *et al*. (2018) showed that amongst cancer patients who were screened as significantly clinically distressed (54% of their sample), only one-quarter intended to use the psycho-oncology service, while one-third were ambivalent and 42% reported no intention. Ambivalent patients reported higher distress than patients with no intention but showed significantly lower uptake behaviour than patients with an intention, emphasizing fears and uncertainties, while patients with clear intentions emphasised knowledge, attitudes and coping concepts. Four months later, 23% had utilised the psycho-oncology service.

Increasingly, an effective distress screening protocol is recognised as a multilevel intervention necessitating change at the system, institutional, provider and patient level to ensure that patients who would benefit from psychosocial support and treatment are appropriately referred and treated (Ehlers *et al*., 2019). Consistent with this, in the updated guidelines, the previous ‘recommendations’ for implementation of the guidelines have been revised and are now explicit principles of implementation aimed at promoting comprehensive screening protocols. They begin by emphasizing the American College of Surgeon's Commission on Cancer's accreditation standard for screening all cancer patients for psychosocial distress (Wagner *et al*., 2013; Pirl *et al*., 2014) and making the appropriate referrals. Whereas the previous text encouraged the creation of an interdisciplinary work group or cancer committee, the new principles delineate the specific disciplines that should be represented on the committee: physician champions, nurses, psychologists, information technology experts, administrative leadership, social workers and chaplains. Rather than suggesting multicentre trials exploring brief screening instruments and piloting treatment guidelines, the principles advocate a small-scale pilot programme to test the comprehensive screening protocol before attempting larger-scale implementation. The principles identify specific aspects to consider: existing resources, current workflows, available information technologies, site-specific cut-off scores (or specific problems), timing and frequency of screening, thresholds for generating referrals, development of response algorithms with alerts and processes for communicating results to critical oncology and psychosocial team members, triggering of appropriate referrals, the inclusion of results into the medical record and tracking of responses to both referrals and interventions to treat distress. Whereas the previous recommendations encouraged efforts to educate staff, patients and families about distress management, the principles of implementation move well beyond this to explicating the need for institutional leadership support and the identification of key stakeholders who will facilitate the implementation of comprehensive screening protocols. Similarly, while continuous quality improvement projects related to management of distress were encouraged, the principles of implementation ask institutions to consider making distress screening a measurable quality metric. Taken as a whole, these principles reflect the current focus on moving beyond routine distress screening to integrating comprehensive screening protocols with a strong emphasis on referral and evidence-based treatment and interventions into the routine clinic care of cancer patients with distress.

A recent survey supports incremental progress in NCCN institutions' efforts to adopt and implement the guidelines, especially with respect to referral and treatment of distressed patients (Donovan *et al*., 2019). The majority of respondents reported having comprehensive screening protocols with routine tracking of clinical contacts, referrals and rates of protocol adherence.

However, a recent Cochrane Review (Schouten *et al*., 2019) concluded that the effectiveness of screening cancer patients' psychosocial well-being and care needs is not clearly supported. The review notes the heterogeneity across studies and calls for more uniformity in outcomes and reporting, the use of intervention description guidelines, and further improvement of methodological certainty in studies, combining subjective patient-reported outcomes with objective outcomes. There is also evidence that patients with high levels of distress who are properly referred and who receive psychological intervention benefit from screening while those with mild distress do not (Sanjida *et al*., 2018). The lack of full-scale implementation to date highlights the need to manage institutional barriers such as insufficient resources and staff and competing demands (Knies *et al*., 2019). To overcome these barriers, cancer care clinicians require training and support to develop and implement psychosocial distress screening programmes in order to be successful in overcoming institutional barriers and meeting the mandate (Ercolano *et al*., 2018).

## Conclusions

Clinical experience and standards of care appear to have resulted in greater guidelines adoption and implementation. Considerable opportunities for improvement remain. These are reflected in the findings that only about a quarter of those surveyed are screening all patients for distress, specific self-report measures being used are many and varied, standardisation is lacking with respect to when patients are screened and rescreened, there is considerable variability across institutions regarding services, resources and staff devoted to triaging and treating distressed patients, and data on the effectiveness of existing comprehensive screening protocols are limited. Similar results have been reported in Europe (Götz *et al*., 2019). There is still work to be done on the fundamental aspects of distress screening, and the need to optimise referrals and ensure the appropriate treatment of patients is pressing. Substantive questions also remain. These include how screening for distress differs from biomedical screening and intervention, how the construct, context and trajectory of distress may complicate efforts to improve patient outcomes (Salmon *et al*., 2015; Jacobsen and Norton, 2019; Palmer, 2019), the role of the patients' perspective (assessment remains dependent on patients' decisions about whether to disclose distress), and the transition from a diagnostic to a public health framework for screening. Such questions are all part of an ongoing debate.
